# Monocyte to High-Density lipoprotein and Apolipoprotein A1 Ratios: Novel Indicators for Metabolic Syndrome in Chinese Newly Diagnosed Type 2 Diabetes

**DOI:** 10.3389/fendo.2022.935776

**Published:** 2022-07-14

**Authors:** Wei Wang, Zhe Yuan Chen, Xiu Li Guo, Mei Tu

**Affiliations:** Longyan First Affiliated Hospital of Fujian Medical University, Longyan, China

**Keywords:** monocyte to high-density lipoprotein ratio, monocyte to apolipoprotein A1 ratio, metabolic syndrome, newly diagnosed type 2 diabetes, optimal cut-off value

## Abstract

**Objective:**

Increasing evidence highlighted that chronic inflammation involved in the development of metabolic syndrome (MetS) and Type 2 diabetes mellitus (T2DM). This prospective study was aimed to assess the association between MetS and novel pro-inflammatory indicators like monocyte–to–high-density lipoprotein and monocyte–to–apolipoprotein A1 ratios (MHR and MAR) in Chinese newly diagnosed T2DM.

**Method:**

A total of 605 Chinese newly diagnosed T2DM with complete and available data were enrolled in this study. Demographic and anthropometric information were collected. Laboratory assessments were determined by standard methods. MetS was based on the Chinese Diabetes Society definition. Multiple binomial logistic regression model was used to estimate the independent variables of MHR and MAR for MetS. Receiver operating characteristic (ROC) curve was conducted to assess the optimal cutoff value of MHR and MAR in identifying MetS.

**Results:**

Overall, the prevalence of MetS was 60.2%. The correlation analysis showed that MHR and MAR were closely correlated with metabolic risk factors like body mass index, waist circumference, triglycerides, high-density lipoprotein cholesterol, systolic blood pressure, diastolic blood pressure, uric acid, and insulin resistance. MHR and MAR were also significantly associated with higher odds of MetS after adjustment for other confounders, the odds ratios (ORs) (95%CI) were 1.50 (1.14–1.97) and 2.26(1.79–2.87) respectively. Furthermore, MHR and MAR were also seemed to have higher area under the curve (AUC) for MetS than ApoA1 and monocyte alone from the ROC curve analysis (*P* < 0.05). The AUCs of MHR and MAR identifying MetS were 0.804 (95% CI: 0.768–0.839) and 0.840 (95% CI: 0.806–0.873) respectively (*P* < 0.001). The optimal cutoff values of MHR and MAR were 3.57 × 10^8^/mmol (sensitivity: 76.1%, specificity: 73.4%) and 3.95 × 10^8^/g (sensitivity: 79.7%, specificity: 84.6%), respectively.

**Conclusions:**

MHR and MAR were significantly associated with MetS. These two novel pro-inflammatory indicators may be useful markers for MetS in Chinese newly diagnosed T2DM.

## Introduction

Type 2 diabetes mellitus (T2DM) is a kind of metabolic disease characterized by chronic hyperglycemia that often accompanied with other metabolic disorders like obesity, hypertension, and hyperlipidemia. Metabolic syndrome (MetS) has gradually become an increasing worldwide health problem that was associated with increased cardiovascular disease (CVD), stroke, and T2DM (1). MetS is closely correlated with T2DM, and epidemiological survey reported the prevalence of MetS was up to 68.1% in Chinese T2DM (2). Chronic low-concentration inflammation, cellular dysfunction, and oxidative stress participate in the occurrence and development of T2DM and MetS (3); the non-invasive detection index of “chronic low concentration inflammatory statues” can be an effective marker for in T2DM with MetS.

Circulating monocyte is a cluster of blood cell modulated by immune factors including tumor necrosis factors alpha (TNF-α) and Toll-like receptor (TLR) 2, TLR4, and TLR8 ligands that can interact with thrombocytes and endothelial cells, resulting in exaggerated inflammation and increased oxidative stress (4–6). These biological features provided a basis for monocyte involving in development of systematic inflammation disease like MetS, T2DM, and CVD (7). High-density lipoprotein (HDL-c) is considered as “good cholesterol” that can bind lipid molecules such as triglyceride (TG) and cholesterol and participate in the cholesterol clearance, resulting in decreased CVD risk (8). Monocyte–to–HDL-c ratio (MHR) was also recognized as indicators of oxidative stress and systemic inflammation, which has been identified as a predictive marker for some disease, such as CVD, polycystic ovarian syndrome (PCOS), and Parkinson’s Disease (9–11). Besides fewer studies reported the predictive value of MHR for MetS in patients with PCOS (10, 12), there was no study that has focused on the potential ability of MHR for MetS in newly diagnosed T2DM. Apolipoprotein A1 (ApoA1) is a constituent of HDL-c produced by liver that participates in the process of peripheral cholesterol reverse transportation to the liver, which was also considered as protective proteins in CVD (13). Despite numerous studies have confirmed that the ratio of apolipoprotein B (ApoB) to ApoA1 is significantly correlated with MetS, no study has put insights to the association between monocyte to ApoA1 ratio (MAR) and MetS. Thereby, this prospective study was aimed to assess the association between MetS and novel pro-inflammatory indicators MHR and MAR in Chinese newly diagnosed T2DM, further evaluating the ability of MHR and MAR in identifying MetS.

## Study Design and Methods

### Study Design and Participants

**Figure 1:**
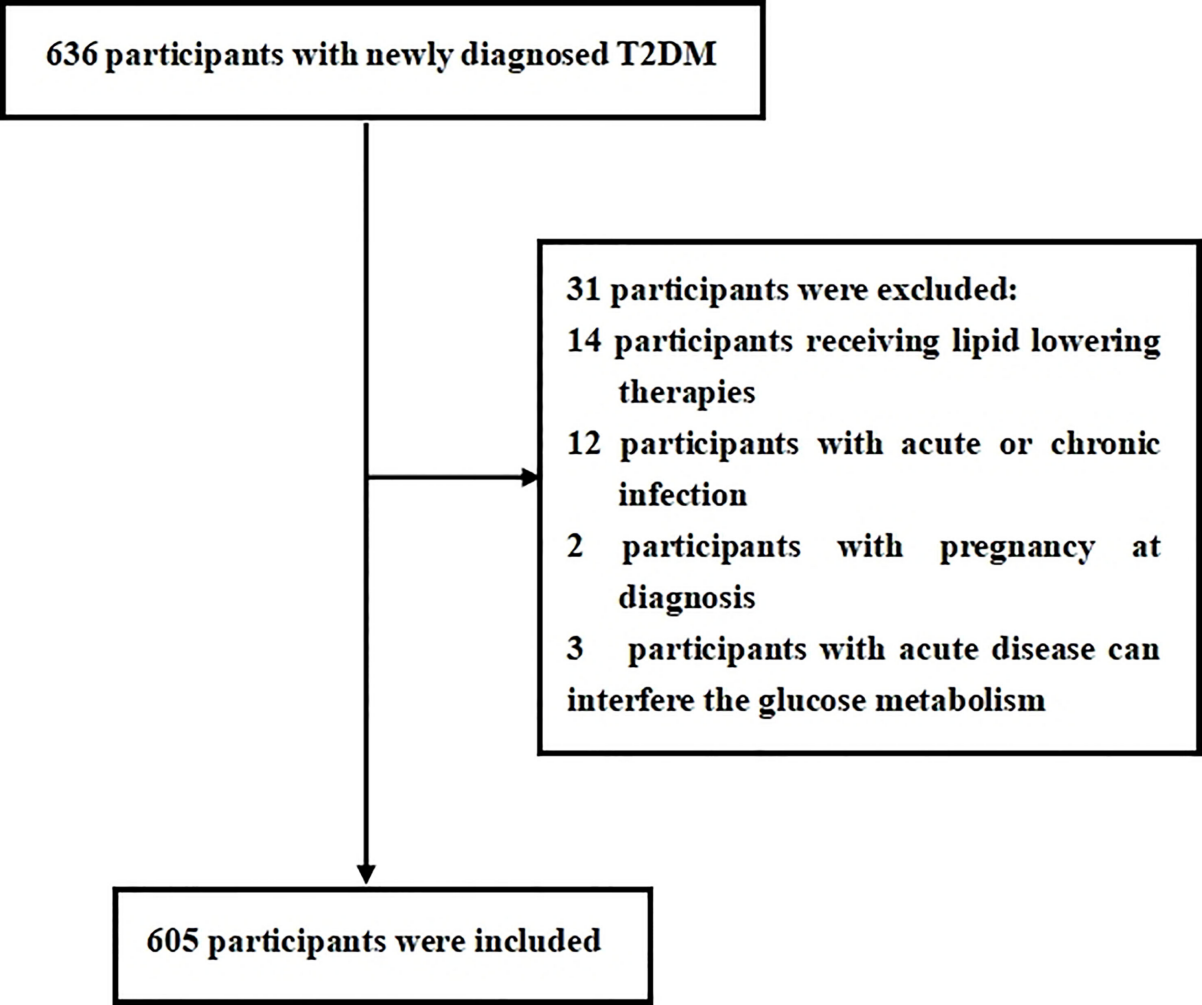
Flow diagram of the participants excluded and included in this study.

This cross-sectional study was consecutively conducted with newly diagnosed T2DM from the Department of Endocrinology at Longyan First Affiliated Hospital of Fujian Medical University who fulfilled the study criteria between January, 2021 and December, 2021. The T2DM was defined according to the World Health Organization (WHO) 2019 criteria: (1) fasting plasma glucose ≥126 mg/dl or 2-h postprandial ≥200 mg/dl during oral glucose tolerance test (OGTT) or HbA1C ≥6.5% or participants with classic symptoms of hyperglycemia or hyperglycemic crisis with random plasma glucose ≥200 mg/dl and (2) with negative diabetic autoimmune antibodies and excluded other specific types of diabetes. Previous unknown hyperglycemia status and c were considered as newly diagnosed T2DM. Participants were excluded if they met the following criteria: (1) presence of acute diseases that can interfere glucose metabolism; (2) presence of acute or chronic infection, obvious liver or renal dysfunction, anemia, hemolytic diseases, and bleeding that can interfere circulating monocyte count; (3) treatment with medications that can interfere circulating monocyte count; (4) currently receiving lipid-lowering therapies; (5) presence of secondary hypertension or a history of tumors; and (6) unwillingness to participate in this study. In this study, we estimated the sample size according to the requirement of multiple binomial logistic regression model; 12–14 variables may be put into the logistic regression model according to the principle of 5–10 events per variable, and the prevalence of MetS is about 50%–70% in newly diagnosed T2DM. Thus, we planned a sampling size of 500–600 participants (2). Overall, a total of 636 participants were screened. Among them, 605 participants meeting the inclusion and exclusion criteria were enrolled in this study. The flow diagram of excluded and included participant was presented in **Figure 1**. All procedures were conducted in accordance with the Declaration of Helsinki. This study was approved by the ethical committee of Longyan First Affiliated Hospital of Fujian Medical University (LY-2020-088). All participants enrolled in the study provided informed consent.

### Anthropometric and Laboratory Assessments

Demographic information was collected by trained interviewers through a standard questionnaire and also obtained by a review of medical records and laboratory data, including gender, age, and history of diseases that can interfere circulating monocyte, current or prior use of drugs, smoking, and drinking. Participants that smoke more than four cigarettes a week for at least 6 months continually or accumulative were considered as smoking according to standardized methodological recommendations of WHO for smoking surveys (14). Participants that drink more than once a year were considered as drinking according to global burden of disease study (15). Physical examination was conducted by the research nurses, including height, weight, waist circumference (WC), and blood pressure (BP). Participants wear hospital gowns and bare feet. Height and weight were measured to the nearest 0.1 cm and 0.1 kg, respectively. Weight was measured using the gauges real-time load cell, and height was measured using the gauges ultrasonic probe. Body mass index (BMI) was calculated as the weight divided by the square of height (kg/m^2^). WC was measured at the natural depression between the iliac crest and 10th rib, which should be the narrowest part of the abdomen. Systolic and diastolic BP (SBP and DBP) were measured by an electronic sphygmomanometer with an appropriate cuff size after the participants take a rest for more than 5 min on at least three different occasions; the mean of three measurements was calculated as final BP.

Laboratory assessments were measured by standard methods using fasting venous blood samples that were taken between 8:00 a.m. and 9:00 a.m. after fasting overnight. Blood samples were taken into standardized tubes containing dipotassium ethylenedinitrilotetraacetic acid. Serum levels of the following variables were determined: creatinine, alanine aminotransferase (ALT), uric acid (UA), fasting blood glucose (FBG), serum insulin, HbA1c, diabetic autoimmune antibodies (GADA, IAA, and ICA), HDL-c, low-density lipoprotein (LDL-c), TGs, ApoA1, high-sensitivity C-reactive protein (hs-CRP), and complete blood count. Biochemical indexes were measured by an auto-biochemical analyzer (Roche Diagnostics Corporation). ApoA1 levels were measured by the polyethylene glycol–enhanced immunoturbidimetric assay (Maker, Chengdu, China). HbA1c was evaluated by high-performance liquid chromatography with a D10 set (Bio-Rad). Complete blood count was obtained using the Coulter LH 780 Analyzer (Beckman Coulter Ireland, Galway, Ireland). Homeostasis model assessment (HOMA-IR) was used to assess insulin sensitively. HOMA-IR was calculated with the following formula: fasting serum insulin (µU/ml) × fasting plasma glucose (mmol/l)/22.5 (16). MHR or MAR was calculated with the following formulas: the monocyte count divided by HDL-c or ApoA1 level.

### Definition of Metabolic Syndrome

Participants were diagnosed with MetS according to Chinese diabetes guideline for MetS management (17). Participants that met three or more of the following criteria are considered to have MetS: (1) abdominal obesity: WC≥90 cm in men or ≥85 cm in women; (2) hyperglycemia: FBG ≥6.1 mmol/L or OGTT 2-h blood glucose≥7.8 mmol/L or previously diagnosed diabetes with treatment; (3) hypertension: BP ≥130/85 mmHg or currently under antihypertension therapy; (4) fasting TGs ≥1.70 mmol/L; and (5) fasting HDL-c <1.04 mmol/L. All participants in this study should fulfill the criteria for hyperglycemia and diagnosed as newly diagnosed T2DM.

### Statistical Analysis

Data were analyzed by using the SPSS 23.0 software (SPSS Inc. IBM). Descriptive data are expressed as means ± standard deviation (SD). Discrete variables were summarized in frequency tables (N, %). Participants were divided into three groups based on tertiles of MHR and MAR. Statistical differences among groups were performed with one-way analysis of variance (ANOVA) followed by Tukey’s test for multiple comparisons. Chi-squared (χ2) test or Fisher’s exact test was used for comparison of categorical variables. Correlation between MHR, MAR, and metabolic parameters was evaluated by Pearson’s or Spearman’s correlation analysis. Multiple binomial logistic regression model was used to estimate the independent variables of MHR and MAR for MetS after adjusting for other covariates. The receiver operating characteristic (ROC) curves were used to assess the identifying value of MHR and MAR for MetS in newly diagnosed T2DM. Optimal cutoff value was based on the greatest value of the Youden’s index. A two-tailed value of *P* < 0.05 was considered statistically significant.

## Results

Overall, a total of 605 newly diagnosed T2DM with complete and available data were included in the final analysis. Clinical and laboratory characteristics of participants were summarized in **Table 1**. Among them, 304 (50.2%) participants were men. The prevalence of MetS was 60.2% with a mean age of 53.4 ± 7.5 years. The MetS group was more likely to have hypertension as compared with the non-MetS group (*P <* 0.05). The BMI, WC, TG, SBP, DBP, UA, HOMA-IR, serum insulin, monocyte count, MAR, and MHR were significantly higher, whereas HDL-c and ApoA1 were significantly lower in the MetS group than the non-MetS group (*P <* 0.05). Moreover, the MHR and MAR were also calculated and divided into three tertiles. Clinical and laboratory characteristics of participants based on tertiles of MHR and MAR were summarized in **Tables 2**, **3**. Increasing trends were observed in BMI, WC, TG, SBP, DBP, UA, serum insulin, HOMA-IR, and monocyte count across the MHR and MAR tertiles (*P <* 0.05). In addition, decreasing trends were also observed in HDL-c and ApoA1 across the MHR and MAR tertiles (*P <* 0.05). Furthermore, participants in higher tertiles of MHR and MAR groups showed the higher prevalence of MetS and hypertension (*P <* 0.05).

**Table 1:** Clinical and laboratory characteristics of participants.

Variable	Total	Non-MetS (n = 241)	MetS (n = 364)	*P*
Age (year)	53.4 ± 7.5	52.8 ± 7.7	53.8 ± 7.5	0.103
Men, n (%)	304(50.2)	126(52.3)	178(48.9)	0.415
BMI (kg/m^2^)	24.3 ± 3.1	23.1 ± 2.4	25.2 ± 3.0	< 0.001
HbA1c (%)	9.0 ± 1.1	9.0 ± 1.0	9.0 ± 1.2	0.521
WC (cm)	85.7 ± 6.9	82.6 ± 5.0	87.7 ± 7.2	< 0.001
TG (mmol/L)	2.14 ± 1.38	1.47 ± 0.88	2.59 ± 1.47	< 0.001
HDL-c (mmol/L)	1.10 ± 0.24	1.24 ± 0.20	1.01 ± 0.23	< 0.001
LDL-c (mmol/L)	3.48 ± 0.90	3.47 ± 0.88	3.49 ± 0.90	0.625
ApoA1 (g/L)	1.03 ± 0.21	1.14 ± 0.20	0.96 ± 0.19	< 0.001
Monocyte (10^9^/L)	0.41 ± 0.10	0.37 ± 0.09	0.44 ± 0.10	< 0.001
UA (µmol/L)	352.1 ± 85.4	326.1 ± 72.7	370.4 ± 88.9	< 0.001
Creatinine (µmol/L)	70.5 ± 13.2	71.7 ± 13.5	69.7 ± 12.9	0.075
ALT (IU/L)	35.0 ± 9.0	34.9 ± 9.1	35.0 ± 9.0	0.874
SBP (mmHg)	132.0 ± 17.4	123.9 ± 13.4	139.1 ± 17.0	< 0.001
DBP(mmHg)	81.2 ± 9.8	76.8 ± 8.1	84.1 ± 9.8	< 0.001
Insulin (mU/ml)	27.6 ± 11.4	19.0 ± 7.7	33.9 ± 9.5	< 0.001
HOMA-IR	11.5 ± 6.2	8.7 ± 5.4	12.6 ± 6.0	< 0.001
hs-CRP (mg/L)	2.9 ± 0.9	2.9 ± 0.9	3.0 ± 1.0	0.788
Hypertension, n (%)	215(35.5)	37(15.4)	178(48.9)	< 0.001
Smoking, n (%)	218(36.0)	81(33.6)	137(37.6)	0.312
Drinking, n (%)	224(37.0)	79(32.8)	145(39.8)	0.079
MAR (10^8^/g)	4.25 ± 1.69	3.34 ± 1.12	4.86 ± 1.73	< 0.001
MHR (10^8^/mmol)	4.02 ± 1.58	3.09 ± 1.16	4.63 ± 1.53	< 0.001

**Table 2:** Clinical and laboratory characteristics of participants based on tertiles of MHR (10^8^/mmol).

Variable	T1 (<3.12)	T2 (3.12-4.57)	T3 (>4.57)	*P*
Age (year)	53.0 ± 7.5	53.2 ± 7.9	54.1± 7.3	0.33
Men, n (%)	100 (49.5)	102 (50.0)	102 (51.3)	0.937
BMI (kg/m^2^)	22.3 ± 2.2^ab^	24.4 ± 2.2^ac^	26.3 ± 3.0^bc^	< 0.001
HbA1c (%)	9.1 ± 1.2	8.9 ± 1.0	9.0 ± 1.1	0.314
WC (cm)	81.1 ± 4.3^ab^	85.5 ± 5.1^ac^	90.5± 7.4^bc^	< 0.001
TG (mmol/L)	1.26 ± 0.89^ab^	1.89 ± 0.71^ac^	3.30± 1.51^bc^	< 0.001
HDL-c (mmol/L)	1.30 ± 0.21^ab^	1.06 ± 0.17^ac^	0.93 ± 0.20^bc^	< 0.001
LDL-c (mmol/L)	3.38 ± 0.88	3.55 ± 0.90	3.50 ± 0.93	0.156
ApoA1 (g/L)	1.09 ± 0.22^ab^	1.04 ± 0.19^ac^	0.96 ± 0.21^bc^	< 0.001
Monocyte (10^9^/L)	0.32 ± 0.06^ab^	0.41 ± 0.05^ac^	0.51 ± 0.08^bc^	< 0.001
UA (umol/L)	298.8 ± 71.7^ab^	357.2 ± 64.7^ac^	402.8 ± 85.1^bc^	< 0.001
Creatinine (umol/L)	71.6 ± 13.3	70.7 ± 13.9	69.2 ± 12.2	0.185
ALT (IU/L)	35.7 ± 9.9	34.0 ± 7.0	35.4 ± 9.9	0.108
SBP (mmHg)	119.3 ± 13.0^ab^	131.6 ± 15.7^ac^	146.5 ± 10.9^bc^	< 0.001
DBP (mmHg)	77.0 ± 6.1^ab^	79.7 ± 10.9^ac^	87.9 ± 7.8^bc^	< 0.001
Insulin (mU/ml)	16.9 ± 8.1^ab^	26.4 ± 9.8^ac^	34.5 ± 10.3^bc^	< 0.001
HOMA-IR	7.2 ± 5.3^ab^	11.1 ± 4.5^ac^	14.1 ± 6.0^bc^	< 0.001
hs-CRP (mg/L)	2.9 ± 0.9	3.0 ± 1.0	3.1 ± 0.9	0.373
Hypertension, n (%)	28 (13.9)^ab^	56 (27.5)^ac^	131 (65.8)^bc^	< 0.001
Smoking,n (%)	66 (32.7)	75 (36.8)	77 (38.6)	0.439
Drinking,n (%)	72 (35.6)	74 (36.3)	78 (39.2)	0.735
MAR (10^8/^g)	3.07 ± 0.86^ab^	4.03 ± 0.77^ac^	5.67 ± 1.97^bc^	< 0.001
MetS, n (%)	60 (29.7)^ab^	128 (62.7)^ac^	176 (88.4)^bc^	< 0.001

BMI, body mass index; UA, uric acid; TG, triglyceride; HDL-c, high-density lipoprotein cholesterol; LDL-c, low-density lipoprotein cholesterol; ApoA1, apolipoprotein A1; SBP, systolic blood pressure; DBP, diastolic blood pressure; HOMR-IR, homeostasis model assessment insulin resistance; hs-CRP, high-sensitivity C-reactive protein; MHR, monocyte–to–HDL-c ratio; MAR, monocyte-to-ApoA1 ratio. ^a^P < 0.05: T1 vs. T2. ^b^P < 0.05: T1 vs. T3. ^c^P < 0.05: T2 vs. T3.

**Table 3:** Clinical and laboratory characteristics of participants based on tertiles of MAR (10^8^/g).

Variable	T1 (<3.49)	T2 (3.12–4.56)	T3 (>4.56)	*P*
Age (year)	52.7 ± 7.3	54.1 ± 8.2	53.5 ± 7.1	0.199
Men, n (%)	101 (49.8)	95 (47.0)	108 (54.0)	0.371
BMI (kg/m^2^)	23.0 ± 2.4^ab^	24.4 ± 2.4^ac^	25.7 ± 3.4^bc^	< 0.001
HbA1c (%)	9.0 ± 1.1	8.9 ± 1.0	9.1 ± 1.1	0.247
WC (cm)	82.5 ± 5.1^ab^	85.5 ± 5.8^ac^	89.0 ± 7.9^bc^	< 0.001
TG (mmol/L)	1.51 ± 0.87^ab^	1.94 ± 1.02^ac^	2.99 ± 1.67^bc^	< 0.001
HDL-c (mmol/L)	1.25 ± 0.23^ab^	1.10 ± 0.20^ac^	0.96 ± 0.23^bc^	< 0.001
LDL-c (mmol/L)	3.46 ± 0.91	3.48 ± 0.88	3.49 ± 0.93	0.965
ApoA1 (g/L)	1.19 ± 0.20^ab^	1.04 ± 0.13^ac^	0.86 ± 0.16^bc^	< 0.001
Monocyte (10^9^/L)	0.33 ± 0.06^ab^	0.42 ± 0.06^ac^	0.50 ± 0.10^bc^	< 0.001
UA (µmol/L)	318.5 ± 70.3^ab^	356.0 ± 74.1^ac^	384.1 ± 96.9^bc^	< 0.001
Creatinine (µmol/L)	71.8 ± 13.3	70.1 ± 13.1	69.6 ± 13.1	0.176
ALT (IU/L)	34.7 ± 8.8	35.6 ± 8.9	34.6 ± 9.4	0.520
SBP (mmHg)	123.5 ± 14.2^ab^	132.7 ± 13.1^ac^	143.2 ± 14.8^bc^	< 0.001
DBP (mmHg)	76.0 ± 9.9^ab^	81.0 ± 6.9^ac^	86.6 ± 9.4^bc^	< 0.001
Insulin (mU/ml)	19.8 ± 8.9^ab^	26.1 ± 8.7^ac^	33.8 ± 9.9^bc^	< 0.001
HOMA-IR	8.2 ± 4.9^ab^	10.9 ± 5.3^ac^	14.0 ± 6.5^bc^	< 0.001
hs-CRP (mg/L)	2.8 ± 0.9	3.0 ± 1.0	3.0 ± 0.9	0.255
Hypertension, n (%)	34 (16.7)^ab^	67 (33.2)^ac^	114 (57.0)^bc^	< 0.001
Smoking, n (%)	72 (35.4)	71 (35.1)	75 (37.2)	0.777
Drinking, n (%)	77 (37.9)	72 (35.6)	75 (37.2)	0.880
MHR (10^8^/mmol)	2.73 ± 0.82^ab^	3.93 ± 0.93^ac^	5.42 ± 1.53^bc^	< 0.001
MetS, n (%)	53 (26.1)^ab^	128 (63.4)^ac^	183 (91.5)^bc^	< 0.001

BMI, body mass index; UA, uric acid. TG, triglyceride. HDL-c, high-density lipoprotein cholesterol. LDL-c, low-density lipoprotein cholesterol. ApoA1, apolipoprotein A1.SBP, systolic blood pressure. DBP, diastolic blood pressure. HOMR-IR, homeostasis model assessment insulin resistance. hs-CRP, high-sensitivity C-reactive protein. MHR, monocyte–to–HDL-c ratio. MAR, monocyte-to-ApoA1 ratio. ^a^P < 0.05: T1 vs. T2. ^b^P < 0.05: T1 vs. T3. ^c^P < 0.05: T2 vs. T3.

The correlations between MHR, MAR, and metabolic parameters were presented in **Table 4**. The results showed that MHR and MAR were positively associated with BMI, WC, TG, SBP, DBP, UA, HOMA-IR, and monocyte count, whereas MHR and MAR were negatively associated with HDL-c and ApoA1 (*P <* 0.05). In addition, A positive correlation between MHR and MAR was also observed (R = 0.762, *P* < 0.001).

**Table 4:** Correlations between MHR, MAR and metabolic parameters.

Variable	MHR		MAR	
	R	*P*	R	*P*
Age (year)	0.045	0.27	0.035	0.384
HbA1c (%)	0.035	0.397	0.058	0.157
WC (cm)	0.502	< 0.001	0.355	< 0.001
BMI (kg/m^2^)	0.51	< 0.001	0.334	< 0.001
TG (mmol/L)	0.44	< 0.001	0.418	< 0.001
LDL-c (mmol/L)	0.023	0.564	−0.018	0.651
HDL-c (mmol/L)	−0.751	< 0.001	−0.455	< 0.001
ApoA1 (g/L)	−0.281	< 0.001	−0.665	< 0.001
Monocyte (10^9^/L)	0.885	< 0.001	0.749	< 0.001
MAR (10^8^/g)	0.762	< 0.001	NS	NS
MHR (10^8^/mmol)	NS	NS	0.762	< 0.001
UA (µmol/L)	0.505	< 0.001	0.342	< 0.001
Creatinine (µmol/L)	−0.075	0.066	−0.069	0.099
ALT (IU/L)	−0.012	0.764	−0.025	0.547
SBP (mmHg)	0.463	< 0.001	0.462	< 0.001
DBP (mmHg)	0.362	< 0.001	0.436	< 0.001
HOMA-IR	0.321	< 0.001	0.35	< 0.001
hs-CRP (mg/L)	0.016	0.678	0.019	0.713

BMI, body mass index; UA, uric acid; TG, triglyceride; HDL-c, high-density lipoprotein cholesterol; LDL-c, low-density lipoprotein cholesterol; ApoA1, apolipoprotein A1; SBP, systolic blood pressure; DBP, diastolic blood pressure; HOMR-IR, homeostasis model assessment insulin resistance; hs-CRP, high-sensitivity C-reactive protein; MHR, monocyte–to–HDL-c ratio; MAR, monocyte-to-ApoA1 ratio.

To determine independent variables of MHR and MAR for MetS, binomial logistic regression analysis was also performed (**Table 5**). The MHR and MAR were associated with MetS in an unadjusted model (model 0), and the odds ratios (ORs) (95%CI) were 2.50(2.12–2.98) and 3.17(2.57–3.91), respectively. The MHR and MAR were shown to be independently associated with MetS after adjustment for age and gender (model 1), and the ORs (95%CI) were 2.51 (2.11–2.98) and 3.18 (2.57–3.92), respectively. A significant association between MHR, MAR, and MetS was also found after further adjustment for HbA1c, BMI, LDL-c, ApoA1, monocyte, UA, and HOMA-IR (model 2), and the ORs (95%CI) were 2.24 (1.82–2.76) and 2.68 (2.14–3.35), respectively. After further additional adjustment for TG, WC, HDL-c, SBP, and DBP (model 3), the ORs remained significant, and the ORs (95%CI) were 1.50 (1.14–1.97) and 2.26(1.79–2.87), respectively. In addition, the ApoA1 and monocyte count were also associated with MetS in model 0, and the ORs (95%CI) were 0.76 (0.68–0.83) and 2.46 (1.99–3.03), respectively. A significant association between ApoA1, monocyte count, and MetS was also found in model 1, and the ORs (95%CI) were 0.77 (0.68–0.84) and 2.47 (1.98–3.03), respectively.

**Table 5:** Binomial Logistic Regression Analysis adjusted ORs (95% CIs) for the associations between MHR, MAR and the risk of MetS.

Models	MHR		MAR	
	OR (95%CI)	*P*	OR (95%CI)	*P*
Model 0	2.50 (2.12-2.98)	< 0.001	3.17 (2.57-3.91)	< 0.001
Model 1	2.51 (2.11–2.98)	< 0.001	3.18 (2.57–3.92)	< 0.001
Model 2	2.24 (1.82–2.76)	< 0.001	2.68 (2.14–3.35)	< 0.001
Model 3	1.50 (1.14–1.97)	0.004	2.26 (1.79–2.87)	< 0.001

Model 0 was an unadjusted model. Model 1 was adjusted for age and gender. Model 2 was additionally adjusted for HbA1c, BMI, LDL-c, ApoA1, monocyte, UA, and HOMA-IR based on model 1. Model 3 was additionally adjusted for TG, WC, HDL-c, SBP, and DBP based on model 2. BMI, body mass index; WC, waist circumference; HbA1c, glycated hemoglobin; UA, uric acid; TG, triglyceride; HDL-c, high-density lipoprotein cholesterol; LDL-c, low-density lipoprotein cholesterol; ApoA1, apolipoprotein A1; SBP, systolic blood pressure; DBP, diastolic blood pressure; HOMR-IR, homeostasis model assessment insulin resistance; hs-CRP, high-sensitivity C-reactive protein; MetS, metabolic syndrome; MHR, monocyte–to–HDL-c ratio; MAR, monocyte-to-ApoA1 ratio.

**Figure 2:**
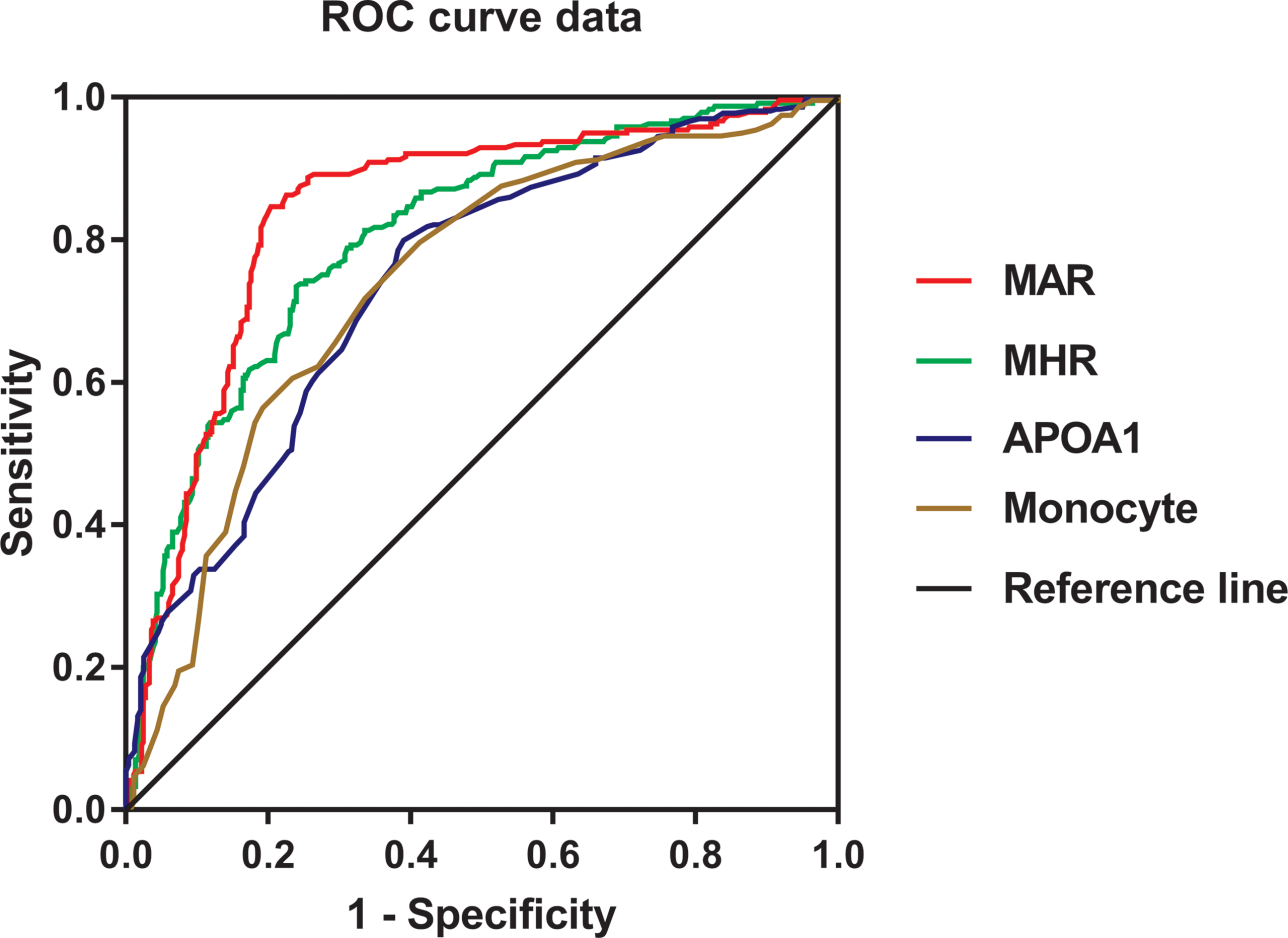
Receiver operating characteristic curves for the cutoff value of MHR, MAR, monocyte, and ApoA1 to identify MetS.

The ROC curve analysis was used to further evaluate the ability of MHR and MAR in identifying MetS. From the ROC curve analysis, the results showed a good identifying value of MHR and MAR for MetS. In addition, MHR and MAR showed higher area under the curve (AUC) in identifying MetS compared with ApoA1 and monocyte alone (*P <* 0.05). MAR also showed the highest AUC in identifying MetS. The AUC of MHR and MAR in identifying MetS was 0.804 (95% CI: 0.768–0.839, *P* < 0.001) and 0.840 (95% CI: 0.806–0.873, *P* < 0.001), respectively (**Figure 2**). The optimal cutoff values of MHR and MAR were 3.57 × 10^8^/mmol (sensitivity: 76.1%, specificity: 73.4%) and 3.95 × 10^8^/g (sensitivity: 79.7%, specificity: 84.6%), respectively (**Table 6**).

**Table 6:** ROC Curve Analysis of MHR and MAR in identifying MetS.

Variables	AUC(95% CI)	Cutoff value	Sensitivity (%)	Specificity (%)
MHR (10^8^/mmol)	0.804 (0.768–0.839)	3.57	76.1	73.4
MAR (10^8^/g)	0.840 (0.806–0.873)	3.95	79.7	84.6
Monocyte (10^9^/L)	0.736 (0.692–0.770)	0.445	52.7	87.6
ApoA1 (g/L)	0.741 (0.701–0.781)	1.14	82.1	56

MetS, metabolic syndrome; ApoA1, apolipoprotein A1; MHR, monocyte–to–HDL-c ratio; MAR, monocyte-to-ApoA1 ratio.

## Discussion

T2DM is a cluster of metabolic disease that often accompanied with MetS at the first diagnosis. Increasing evidence highlighted that inflammation involved in the development of MetS and T2DM. In this cross-sectional study, we mainly assessed the association between MetS and novel inflammatory indicators MHR and MAR in Chinese newly diagnosed T2DM. As expected, the results in the present study demonstrated that MHR and MAR were closely associated with metabolic risk factors. MHR and MAR were also significantly associated with higher odds of MetS after adjustment for other confounders. Furthermore, MHR and MAR were also seemed to have higher AUC value for MetS than ApoA1 and monocyte alone from the ROC curve analysis. These findings indicated that MHR and MAR can be novel markers for MetS in Chinese newly diagnosed T2DM.

In recent years, increasing evidence demonstrated that metabolic disorders can trigger inflammatory responses as a coping mechanism toward metabolic changes, leading to chronic inflammation occur. Thereby, chronic inflammation is widely considered as common denominator in many diseases such as obesity, MetS, T2DM, and CVD (7, 18, 19). The inflammatory process is continuous when chronic inflammation occurs, WBCs play an important role and involved in the process of inflammation that can secrete inflammatory cytokines, which can initiate and upregulate inflammatory responses. In the classification of WBCs, monocyte are produced from bone marrow and accumulated in circulatory system for a few days before migrating and differentiating into macrophages (20), which are known to stimulate the immune system and increase inflammation through releasing inflammatory cytokines like tumor necrosis TNF-ɑ, interleukin-6, and monocyte chemoattractant protein 1 (4, 21). In addition, clinical studies also observed that peripheral total monocyte counts were increased parallel to the clustering of component of MetS in T2DM (22, 23). These biological features provided a basis for monocyte to may be a predictive marker for chronic inflammatory disease like MetS and T2DM. Despite the result in our study suggested that monocyte alone is capable of predicting MetS, the identifying value is not good enough with relatively lower AUC of 0.736. The ratio of monocyte to other anti-inflammatory factors may better reflect inflammatory state and has better identifying value for MetS in newly diagnosed T2DM.

HDL-c is capable of binding to lipid molecules that ensure that it has anti-inflammatory effects, which was also considered as an ideal marker of anti-inflammatory factors. More studies have put insights on the association between ratio of neutrophils, lymphocyte to HDL-c (NHR and LHR) or MHR and systematic inflammatory diseases. Chen et al. reported that NHR and LHR have strong predictive power for MetS in Chinese population (24). MHR was also considered as indicators of oxidative stress and systemic inflammatory disease. Several studies reported that MHR showed a powerful predictive value for chronic inflammatory disease like PCOS (10), peripheral artery disease (25), central retinal artery occlusion (26), Parkinson’s Disease (11), nonalcoholic fatty liver disease (27), and ST-elevation myocardial infarction (28). Furthermore, Jiang et al. found that MHR was significantly related to all-cause and cardiovascular mortality in the general population independent of established risk factors (9). De Matteis et al. reported that MHR was independently correlated with vitamin D deficiency in healthy and metabolic women (29). All clinical findings indicated MHR can be a predictive marker for other kinds of chronic inflammatory disease like MetS. To our expectation, the results in our study showed that MHR was significantly associated with higher odds of MetS and seemed to have a higher predictive value for MetS than monocyte alone, and more studies with enough follow-up should be conducted to further confirm these findings. ApoA1 is another kind of hypothetical markers for anti-inflammation produced by the liver and responsible for peripheral cholesterol transportation and redistribution, which was also well recognized as anti-inflammatory lipid proteins in CVD (13). Previous studies have focused on the association between APOB/ApoA1 and MetS. Several studies observed that APOB/ApoA1 was significantly associated with higher odds of MetS and insulin resistance in Chinese population and PCOS patients (30, 31). To further explore the potential effects of ApoA1 in predicting MetS, we analyzed the association between MAR and MetS. To our surprise, MAR was not only an independent risk factor of MetS but it also showed the highest AUC of 0.840 with 79.7% sensitivity and 84.6% specificity in identifying MetS. These findings indicated that MAR may be a more promising indicator of MetS for Chinese newly diagnosed T2DM, whereas more longitudinal studies compared with other inflammatory indicators are needed to further confirm these findings.

To our knowledge, this is the first study that confirmed the identifying value of MHR and MAR for MetS in Chinese newly diagnosed T2DM. The other strengths of this study adjusted several potential confounding variables in final analysis and included enough sample size that can represent the Chinese newly diagnosed T2DM population. Meanwhile, some limitations need to be mentioned. First, this study was designed as a cross-sectional study without follow-up, and it cannot directly reflect the associations MHR, MAR, and MetS. Second, the studied population is the Chinese newly diagnosed T2DM, and the optimal cutoff values of MHR and MAR may be not applicable to other races.

In conclusion, two novel indicators of MetS for Chinese newly diagnosed T2DM was found in this study. The results showed that MHR and MAR were significantly associated with MetS and seemed to have higher AUC value for MetS than ApoA1 and monocyte alone. All these findings indicated that MHR and MAR could be convenient and reliable predictors to screen for MetS in Chinese newly diagnosed T2DM, whereas more longitudinal studies are needed to further confirm these associations.

## Data Availability Statement

The raw data supporting the conclusions of this article will be made available by the authors, without undue reservation.

## Ethics Statement

The studies involving human participants were reviewed and approved by the ethical committee of Longyan First Affiliated Hospital of Fujian Medical University. The patients/participants provided their written informed consent to participate in this study.

## Author Contributions

WW took charge of the software and contributed to writing— original draft. WW, ZC, XG, and MT conducted the investigation. MT contributed to data curation and writing-editing. All authors contributed to the article and approved the submitted version.

## Conflict of Interest

The authors declare that the research was conducted in the absence of any commercial or financial relationships that could be construed as a potential conflict of interest.

## Publisher’s Note

All claims expressed in this article are solely those of the authors and do not necessarily represent those of their affiliated organizations, or those of the publisher, the editors and the reviewers. Any product that may be evaluated in this article, or claim that may be made by its manufacturer, is not guaranteed or endorsed by the publisher.
